# Humeral retroversion and shoulder rotational mobility in young handball practitioners

**DOI:** 10.1590/1413-785220152306149003

**Published:** 2015

**Authors:** Gustavo Aguiar Quadros, Marcelo Baptista Döhnert

**Affiliations:** 1 Universidade Luterana do Brasil, Physiotherapy Course, Torres, RS, Brazil. Work developed at Universidade Luterana do Brasil, Physiotherapy Course, Torres, RS, Brazil.

**Keywords:** Bone retroversion, Shoulder, Athletic injuries, Photogrammetry

## Abstract

**OBJECTIVE:**

: To evaluate the prevalence of humeral retroversion and rotational mobility (RHH) in young handball practitioners and non-practitioners.

**METHODS:**

: This is a cross-sectional study performed with two groups: the handball group, with 14 female students practicing handball and the control group, with 13 young participants non-practicing pitch sports.

**RESULTS:**

: The handball group presented full rotational movement (FRM) hi-gher than the control group in both the dominant shoulder (p=0.001) and the non-dominant shoulder (p=0.0001). The mobility of active and passive internal rotation was significantly higher in handball players in both shoulders. The handball group presented lower internal rotation range of motion for the dominant shoulder as compared to the non-dominant shoul-der (p=0.001).

**CONCLUSION:**

: Young handball practitioners, des-pite skeletally immature, showed a higher MRT than the control group. The handball group showed loss of internal rotation (medial) on the dominant shoulder as compared to the non--dominant shoulder. Level of Evidence II, Prospective Study.

## INTRODUCTION

Handball is a pitch and contact sport that determines high demands of the shoulder joint, using positions and movements that lead to high risk for ligament, tendon and capsular injuries of this joint.^1^ The handball pitch is a complex and fast split gesture which comprises five stages: progression, passing, arm frame, arm acceleration and deceleration.^2^

The humeral torsion or humeral head retroversion (UHR) is an anatomical feature that exists only in monkeys and hu-mans; it is defined, in anatomy, as the spiral movement of this bone, stabilizing at the end of growth with the closure of the humerus proximal epiphyseal line.^3^ ^4^ It is known that UHR is characterized by increased external rotation range of motion (ROM) of and decreased internal rotation of the shoulder at 90˚ abduction.^5^

In athletes practicing throwing sports such as handball, abduc-tion and external rotation take place, generating adaptations both of soft tissue and bone structure.^4^ Injuries that occur in these sports are related especially to movement.^1^ The loss of internal rotation (medial) of the dominant limb compared to the non-dominant limb of the pitcher is called GIRD (Glenohumeral Internal Rotation Deficit) 6 7 and has been related to an adaptive contracture of the posterior capsule in pitchers.^8^

There are several instruments to evaluate GIRD. Among them, stand out bio-photogrammetry 9 10 and goniometry.^3^ Compu-ted photogrammetry is art, science and reliable information technology used to quantify postural changes through the application of photogrammetric principles to photographic images obtained from body movements, complementing eva-luation for physical therapy diagnosis in different areas.^9^ ^11^ It is a noninvasive assessment resource that not only has advantages in the effectiveness of its clinical application with low cost, but also provides high precision and reproducibility of results.^12^ Another widely used evaluation method is gonio-metry.^13^ The goniometric measurements are used by physical therapists to quantify the limitation of joint angles, to decide the most appropriate therapeutic intervention and to document the effectiveness of this intervention.^13^

This study aimed to evaluate the prevalence of humeral head retroversion and characteristics of rotational shoulder mobility in young handball practitioners.

## MATERIALS AND METHODS

This is a cross-sectional study conducted between September and October 2013. The sample consisted of 27 young wom-en, aged 15.07 ± 1.17 years. The sample was divided into handball practitioners group (n = 14) and non-practitioners (control) group (n = 13).

 Young female, aged between 15 and 17 years, regular hand-ball practitioners and non-practitioners of other pitch sports, with stable vital signs and normal physical condition were included in the study. Girls with a history of shoulder joint in-jury in the last six months, practitioners of other pitch sports, who have had surgery in the neck or upper limbs, general ligamentous laxity and neurological or systemic disease were excluded from the study.

The study was approved by the Research Ethics Committee of the *Universidade Luterana do Brasil* , Torres, RS, Brazil, under protocol number 319 570/2013. Along with the coach and their parents, the study subjects were informed about the research and were asked to sign a Free and Informed Consent Form (FICF) drafted in accordance with the Guidelines and Regu-latory Norms of research involving human subjects from the

*Resolução do Conselho Nacional da Saúde* nº196/96.

The assessment protocol was explained right away and after the parents/legal guardians signed the FICF, volunteers were submitted to evaluation.

### Assessment Protocol

Initially, the young participants of both groups were evalu-ated for their anthropometric aspects. They were weighed in a previously calibrated Mallory(r) (Brazil) digital scale using light clothing and no shoes on. Three measurements were performed, and their median was recorded. Height was mea-sured using a Megaforth(r) (Brazil) self-locking 8m measuring tape. Three measurements were made and their median was recorded. Finally, we calculated the body mass index (BMI) of all study subjects.

The measurement of external and internal rotation ROM of both shoulders (dominant and non-dominant) was performed either passively as actively through goniometry and bio-photogrammetry. Goniometry was performed with the subject lying supine on a stretcher with his shoulder in 90° abduction and elbow flexed at 90°. The examiner stabilized the shoulder at the same time that the active and passive external and internal rotation movements ROM were measured on both dominant and non-dominant sides. The center of the goniometer was positioned in the olec-ranon of the ulna, the fixed arm of the goniometer remained fixed and aligned on the vertical axis to the ground, while the movable arm accompanied the movement aligned to the mid-dle line of the forearm.

For evaluating rotational mobility of the shoulder through bio-photogrammetry, a 7.2 megapixels no-zoom Sony DSC W120^(r)^ (Brazil) digital camera was used. The subject remained lying supine on the table with her shoulder abducted 90° and elbow flexed at 90°. White, spherical 13mm non-reflective surface markers were used, placed on the olecranon and the ulnar styloid process. Images of active and passive ROM of both shoulders were acquired with the shoulder in neutral rotation, maximum external rotation and maximum internal rotation. The camera-subject distance was 2.10 m, with a photographic tri-pod at one meter from the ground. The examiner performed the stabilization of the glenohumeral joint during movement. After-wards, the images were transferred to a computer and analyzed by Corel Draw 9.0(r) software. The maximum active and passive ROM of internal and external rotation of each shoulder was cal-culated, as well as the active total rotational movement (TRM).

### Statistical Analysis

A descriptive analysis of the study variables was carried out with data expressed in frequency, mean and standard deviation. For evaluation of angular measurements between athletes and controls we used the unpaired Student t-test. For evaluation of the dominant and non-dominant shoulder within each group, we used the Pearson correlation test. The significance level for the statistical test was *p* <0.05. We use the SPSS (Statistical Pa-ckage for Social Sciences), version 17.0, as statistical package.

## RESULTS

The sample was divided into two distinct groups: handball group, formed by 14 young female practitioners of competitive handball, aged 15.57 ± 1.16 years, height 163.93 ± 0.06 cm, BMI 23.56 ± 3.31, and the control group, made up of 13 young women from a high school in the city of Torres, RS, Brazil, not practitioners of handball or other pitch sports, aged 14.54 ± 0.96 years, 159.9 ± 0.05 cm height, BMI 23.50 ± 4.28. Both groups were homogeneous regarding age, height, BMI, skin color and dominant upper limb. (Table 1)

The internal rotation mobility of both active and passive shoulders measured by goniometry was significantly higher (p <0.05) in the handball group both for the dominant as non-dominant shoulder. The external rotation was significantly higher in the handball group only in the passive form in the non-dominant shoulder. (Table 2)

When we evaluated the active and passive mobility of external and internal rotation by bio-photogrammetry, the young hand-ball practitioners also showed a significant increase in active internal rotation of the dominant and non-dominant shoulders. The passive ROM of this group was significantly higher only in the non-dominant shoulder. Passive ROM was significantly higher in the handball group in both dominant and non-domi-nant shoulders (p<0.05). (Table 3)

The handball group presented both in goniometry and bio-photogrammetry a significantly lower active and passive in-ternal rotation ROM for the dominant shoulder. In contrast, we observed a significantly higher ROM in passive external rota-tion of the dominant shoulder by goniometry (p <0.05). Bio-photogrammetry showed similar results as goniometry. There was a significantly lower internal rotation ROM of the dominant shoulder (p <0.05). There was no significant difference in TRM between dominant and non-dominant shoulders. (Table 4)

In the control group we found that external rotation was sig-nificantly higher in the dominant shoulder in both active and

**Table 1:**
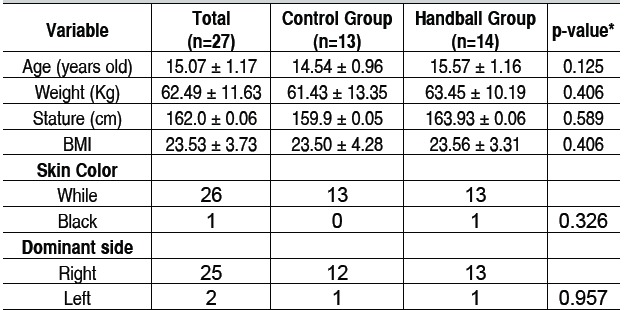
Characterization of the sample

Values expressed in Mean and Standard Deviation. *Chi-square test

passive forms. The internal rotation ROM was higher in the non-dominant shoulder than the dominant shoulder, but only in the passive movement (p <0.05). There was also no sig-nificant difference in TRM comparing the dominant and non-dominant shoulders. (Table 5)

**Table 2:**
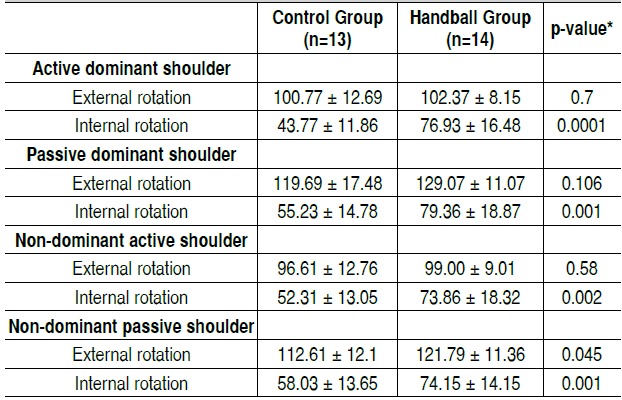
Shoulder range of motion (degrees) assessed by goniometry in young handball practitioners and non-practitioners

Values expressed in Mean and Standard Deviation

**Table 3:**
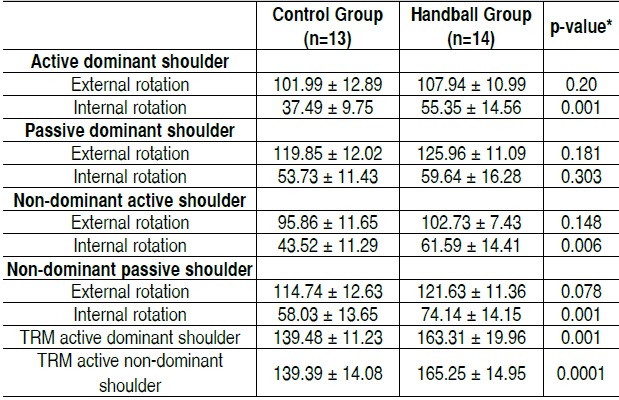
Shoulder range of motion (degrees) assessed by bio-pho-togrammetry in young handball practitioners and non-practitioners.

TRM: Total rotational movement. Values expressed in Mean and Standard Deviation

**Table 4:**
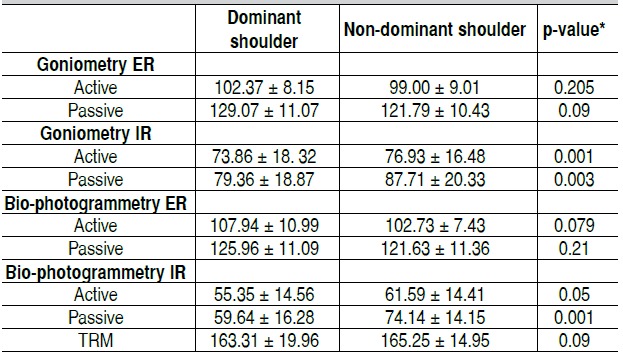
Comparison of active and passive articular range of motion (degrees) between dominant and non-dominant shoulder in young handball practitioners.

ER: External rotation; IR: Internal rotation; TRM: Shoulder active total rotational movement. Values expressed in Mean and Standard Deviation

**Table 5:**
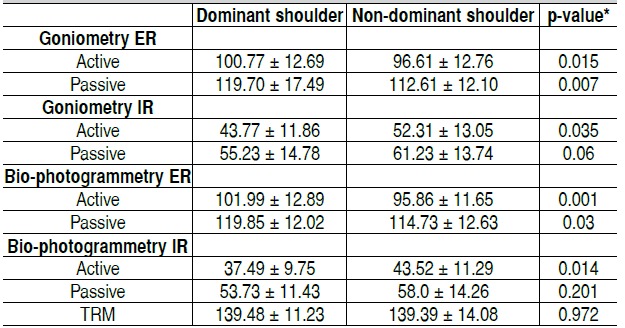
Comparison of active and passive articular range of motion (degrees) between dominant and non-dominant shoulders in young handball non-practitioner.

ER: External rotation; IR: Internal rotation; TRM: Shoulder active total rotational movement. Values expressed in Mean and Standard Deviation

## DISCUSSION

Due to the scarcity of studies evaluating UHR and GIRD in young skeletally immature handball athletes, we sought to in-crease knowledge about the effects generated by the practice of pitching on young women and their possible implications in adulthood.

In this study, we sought to evaluate UHR, represented by GIRD in young handball practitioners and non-practitioners. This con-dition is characterized by the increase in lateral rotation range of motion and reduced medial rotational.^14^ Our sample included young skeletally immature females, with a mean age of 15.07

± 1.17 years. Osbahr et al.,^15^ in their research studied on 19 young male baseball players and suggested that the develop-ment of an increased humeral head retroversion would occur after 11 years old. The authors report that most of this bone growth occurs at the proximal epiphysis after that age.

Levine et al., 16 in a study with 298 players of the Children's Baseball League, stated that the age at which the develop-ment of bone adjustments occurs leading to increased lateral rotation would be between 13 and 16 years old, but the au-thors did not consider the start age of sports practice in these athletes.^15^ In our study we also did not evaluate the mean time of onset of handball practice.

In another study, Murachovsky et al.^4^ assessed 17 male hand-ball players with a mean age of 24 years old all with 12 years of training, on average. Athletes who began to play before 10 years old had higher retroversion. The authors found that there is a statistical relationship between the increased retroversion with increased lateral rotation.

When we evaluated the results of our study, we observed that in the handball group there was no difference in passive and ac-tive external rotation between the dominant and non-dominant shoulders. In contrast, we found a significant loss of internal rotation. The average passive external rotation in the handball group in our study was 129° measured by the goniometry and 126° by bio-photogrammetry. A study by Nodehi-Moghadam et al.^17^ showed no significant difference in internal rotation be-tween athletes and non-athletes. However, external rotation was significantly higher in the athlete group. Brown et al.^18^ found in 19 professional pitcher athletes 141° ER on average in 90°ab-ducted shoulders. The authors also found a ROM 9° higher for non-dominant shoulders.

Recently, Bigliani et al.^19^ reported in their study that the ER of the dominant shoulder measured in 90° abducted shoulders resulted in 118° in pitchers, while the average was 108° for non-dominant shoulders in non-pitchers. The control group in our study aver-aged 120° ER by both goniometry and bio-photogrammetry. The handball group showed a significantly lower active and passive in-ternal rotation of the dominant shoulder. In contrast, a significantly higher ROM passive external rotation of the dominant shoulder was obtained in the handball group. Luna et al.^1^ evaluated 21 athletes of the Brazilian male handball team and found that the athletes showed no significant IR ROM decrease between shoul-ders. However, the study conducted by Chant et al.^20^ studying 25 subjects, 19 baseball athletes and six controls found that the highly competitive players had a higher UHR in their dominant arm. Pascoal and Tainha 21 did not find higher external rotation ROM in water polo players as compared to the control group.

In our study, despite the young handball practitioners showed a decreased internal rotation in the dominant shoulder, the TRM of the handball group was significantly higher than the con-trol group's. Wilk et al.^22^ evaluated the TRM of baseball play-ers shoulders. The authors reported that TRM of professional pitchers shoulder should be up to 5° higher than non-dominant shoulders. A TRM arc greater than 5° may be a contributing fac-tor to possible injuries in pitcher athletes.^22^A study conducted by Yamamoto et al.^5^ with junior baseball players showed a significant difference in the amplitude of TRM between the dominant and non-dominant shoulders.

The TRM in our study showed no significant difference be-tween dominant and non-dominant shoulder in both groups.

However, it was significantly higher in the handball group as compared to the control group. Wilk et al.^23^ enumerate several causes that lead to increased external rotation and loss of internal rotation. These include bone adaptation, shortening of the posterior capsule and shortening of the posterior portion of the rotator cuff and posterior deltoid. Burkhart et al.^8^ described initially the GIRD as the loss of internal rotation amplitude in the pitcher shoulder. Kibler et al.^24^ reported the GIRD as a loss equal to or higher than 18° of the internal rotation of the shoulder pitch as compared to its contralateral side. That GIRD may be the main cause of shoulder pain and disability during pitch. We believe that these bone and capsule muscle adaptations leading to GIRD were not observed in the hand-ball group due to the skeletally immatureness of the sample and low handball practice time.

## CONCLUSION

Young women in early stages of handball training sports sho-wed a significant loss of internal rotation in the dominant shoul-der as compared to the contralateral shoulder, characterizing GIRD. However, the TRM was significantly higher in the handball group as compared to the control group.

The results suggest that stretching of the posterior capsule may be one of the aspects to be addressed in the prevention of further injuries in young handball practitioners. We suggest that further studies on this topic are carried out to increase the scientific knowledge on shoulder rotational mobility cha-racteristics of young handball practitioners and its possible implications in adulthood. 

## References

[B1] Luna NMS, Nogueira GB, Saccol M, Leme L, Garcia MC, Cohen M (2009). Ampli-tude de movimento rotacional glenoumeral por fotogrametria computadori-zada em atletas da seleção brasileira de handebol masculino. Fisioter Mov.

[B2] Montes FA, Dezan DB, Santos DC, Martini E, Zimmerman CA, Gomes SC (2012). Análise tridimensional do arremesso com apoio no handebol. UNOPAR Cient Ciênc Biol Saúde.

[B3] Leal HP, Checchia SL. (2006). A retroversão da cabeça do úmero: Revisão da li-teratura e mensuração em 113 úmeros de cadáveres. Rev Bras Ortop.

[B4] Murachovscy J, Ikemoto RY, Nascimento LGP, Bueno RS, Coelho JA, Komeçu MT, (2007). Avaliação da retroversão da cabeça do úmero em jogadores de handebol. Acta Ortop Bras.

[B5] Yamamoto N, Itoi E, Minagawa H, Urayama M, Saito H, Seki N, (2006). Why is the humeral retroversion of throwing athletes greater in dominant shoulders than in nondominant shoulders?. J Shoulder Elbow Surg.

[B6] Myers JB, Laudner KG, Pasquale MR, Bradley JP, Lephart SM. (2006). Glenohumeral range of motion deficits and posterior shoulder tightness in throwers with pa-thologic internal impingement. Am J Sports Med.

[B7] Lintner D, Mayol M, Uzodinma O, Jones R, Labossiere D (2007). Glenohumeral in-ternal rotation deficits in professional pitchers enrolled in an internal rotation stretching program. Am J Sports Med.

[B8] Burkhart SS, Morgan CD, Kibler WB (2003). The disabled throwing shoulder: spec-trum of pathology Part I: pathoanatomy and biomechanics. Arthroscopy.

[B9] Rodrigues ACC, Romeiro CAP, Patrizzi LJ (2009). Avaliação da cifose torácica em mulheres idosas portadoras de osteoporose por meio da biofotogrametria computadorizada. Rev Bras Fisioter.

[B10] Lunes DH, Castro FA, Moura IC, Oliveira AS, Bevilaqua-Grossi D (2005). Confiabi-lidade intra e interexaminadores e repetibilidade da avaliação postural pela fotogrametria. Rev Bras Fisioter.

[B11] Baraúna MA, Duarte F, Sanches HM, Canto RST, Malusá S, Campelo-Silva CD, (2006). Avaliação do equilíbrio estático em indivíduos amputados de membros inferiores através da biofotogrametria computadorizada. Rev Bras Fisioter.

[B12] Sanchez HM, Barreto RR, Baraúna MA, Canto RST, Morais EG, (2008). Avaliação postural de indivíduos portadores de deficiência visual através da biofotogra-metria computadorizada. Fisioter Mov.

[B13] Marques AP (2003). Manual de goniometria.

[B14] Reinold MM, Gill TJ, Wilk KE, Andrews JR. (2010). Current concepts in the evaluation and treatment of the shoulder in overhead throwing athletes, part 2: injury prevention and treatment. Sports Health.

[B15] Osbahr DC, Cannon DL, Speer KP (2002). Retroversion of the humerus in the throwing shoulder of college baseball pitchers. Am J Sports Med.

[B16] Levine WN, Brandon ML, Stein BS, Gardner TR, Bigliani LU, Ahmad CS (2006). Shoulder adaptive changes in youth baseball players. J Shoulder Elbow Surg.

[B17] Nodehi-Moghadam A, Nasrin N, Kharazmi A, Eskandari Z (2013). A Comparative study on shoulder rotational strength, range of motion and proprioception between the throwing athletes and non-athletic persons. Asian J Sports Med.

[B18] Brown LP, Niehues SL, Harrah A, Yavorsky P, Hirshman HP (1988). Upper extremity range of motion and isokinetic strength of the internal and external shoulder rotators in major league baseball players. Am J Sports Med.

[B19] Bigliani LU, Codd TP, Connor PM, Levine WN, Littlefield MA, Hershon SJ (1997). Shoulder motion and laxity in the professional baseball player. Am J Sports Med.

[B20] Chant CB, Litchfield R, Griffin S, Thain LM (2007). Humeral head retroversion in com-petitive baseball players and its relationship to glenohumeral rotation range of motion. J Orthop Sports Phys Ther.

[B21] Pascoal AG, Tainha C. (2006). Alterações no padrão de rotação externa e abdução horizontal do braço em jogadoras de pólo aquático. Re(habilitar).

[B22] Wilk KE, Meister K, Andrews JR (2002). Current concepts in the rehabilitation of the overhead throwing athlete. Am J Sports Med.

[B23] Wilk KE, Hooks TR, Macrina LC (2013). The modified sleeper stretch and modified cross-body stretch to increase shoulder internal rotation range of motion in the overhead throwing athlete. J Orthop Sports Phys Ther.

[B24] Kibler WB, Kuhn JE, Wilk K, Sciascia A, Moore S, Laudner K, (2013). The disa-bled throwing shoulder: spectrum of pathology-10-year update. Arthroscopy.

